# Penn State Emergency Medicine CarES: Care‐partner Evaluation and Sourcing in the ED

**DOI:** 10.1002/alz70858_103880

**Published:** 2025-12-25

**Authors:** Rollin Wright

**Affiliations:** ^1^ The Pennsylvania State University Milton S. Hershey Medical Center, Hershey, PA, USA

## Abstract

Sustaining transitions of care of people living with dementia (PLWD) from emergency departments (ED) to home is a GEAR 2.0‐ADC research priority. The key to successful transitions of care for PLWD is the care‐partner. 11 million unpaid care‐partners support PLWD in the United States. Most report high levels of stress. Over 40% of PLWD discharged home from the ED return within 30 days. We wondered if care‐partner stress impacted return visits to the ED. The purpose of our pilot, which is currently underway, is to measure the association between the stress reported by care‐partners of community‐dwelling PLWD being discharged to home from our ED and the rate of recurring visits to the ED. Initially, we used a variety of promotional strategies to encourage ED clinicians to identify potential participant dyads. But we learned quickly that our team could not rely on our colleagues to remember and help recruit for our study. Eligible participants are identified as having cognitive impairment either by preexisting diagnosis or via a cognitive assessment screen completed with the care partner of a person who shows signs of cognitive impairment during ED evaluation. Further, to be eligible, the disposition had to be discharge to home from the ED. This requirement has been tricky for a number of reasons. Potential participants may leave quickly after the disposition decision has been made or, conversely, the ED provider reverses course and decides to admit them. Successfully recruited participants complete a screen for caregiver stress, a stress thermometer, and list 3 “care‐giving things” that cause the most stress. In this session, we will share lessons learned about recruiting in this setting, the last minute “soft” admissions to the hospital, and having a difficult conversation about the stress of caregiving.

